# 1913. Baltimore City Health Department COVID-19 Community-based Testing Results

**DOI:** 10.1093/ofid/ofac492.1540

**Published:** 2022-12-15

**Authors:** Shamola Dye, Isabella Agnes, Darcy Phelan-Emrick, Adena Greenbaum

**Affiliations:** Baltimore City Health Department, Aberdeen, Maryland; Baltimore City Health Department, Aberdeen, Maryland; Baltimore City Health Department, Aberdeen, Maryland; Baltimore City Health Department, Aberdeen, Maryland

## Abstract

**Background:**

The Baltimore City Health Department (BCHD) began community-based COVID-19 testing in response to the pandemic on April 21, 2020. The purpose was to provide access to COVID-19 testing to underserved communities. BCHD designed the program to accommodate transportation limitations, limited-access to internet and phones, and non-English speakers. BCHD has continued hosting up to five community-based testing sites per week. This analysis examines the test results of BCHD’s COVID-19 community-based testing program to date.

**Methods:**

Patients completed an intake form, which included demographic information, at the testing event, prior to providing their specimen for COVID-19 testing. For this analysis, patient demographics and test results were analyzed using REDCap software.

**Results:**

Total test volume for year 2020 (4/21/2020 -12/30/2020) was 15,839, year 2021 (1/5/2021- 12/30/2021) was 13,087, and year 2022 (1/4/2022 - 4/1/2022) was 2,261. Average percent positivity for year 2020 was 9%, year 2021 was 7%, and year 2022 to date was 6%. Patient ethnicity was Non-Hispanic 85.5%, Hispanic 13.5%, and Did not respond/Don’t know 1.0%. Race was Black or African America 61.1%, White 25.0%, Asian 2.8%, American Indian/Alaska Native 0.1%, Native Hawaiian or Other Pacific Islander 0.1%, and Unknown 10.7%. Total positive COVID-19 results among Black/African Americans in 2020, 2021, and 2022 respectively were 1567 (12.8%), 1177 (15.1%), and 218 (22.1%). Total positive COVID-19 results among White in 2020, 2021, and 2022 respectively were 541 (10.1%), 441 (12.1%), and 60 (15.1%) respectively. Total positive COVID-19 results among Asians in 2020, 2021, and 2022 were 63 (20.9%), 47 (11.0%), and 4 (25.5%) respectively. Total positive COVID-19 results among Native Hawaiian or Other Pacific Islander in 2020, 2021, and 2022 respectively were 4 (16.7%), 4 (20.0%), and 2 (0.0%) respectively. Total positive COVID-19 results among American Indian/Alaskan in 2020, 2021, and 2022 were 3 (13.6%), 1 (7.1%), and 2 (66.7%) respectively.

**Conclusion:**

BCHD’s community-based testing program has completed over 31,000 tests to date. COVID-19 percent positivity among patients showed a decline across the three years. This analysis demonstrates how a local health department can provide testing to the communities.

**Disclosures:**

**All Authors**: No reported disclosures.

